# Prevalence of fractures in women with rheumatoid arthritis and/or systemic lupus erythematosus on chronic glucocorticoid therapy

**DOI:** 10.1186/s12891-015-0733-9

**Published:** 2015-10-15

**Authors:** Maria Luz Rentero, Encarna Amigo, Nicolas Chozas, Manuel Fernández Prada, Lucia Silva-Fernández, Miguel Angel Abad Hernandez, Jose Maria Rodriguez Barrera, Javier del Pino-Montes, on behalf of the GHDP study group

**Affiliations:** Eli Lilly and Company, C/ Rey Francisco 11, 28008 Madrid, Spain; Hospital de Lugo, San Cibrao, s/n, 27003 Lugo, Spain; Hospital Puerta del Mar, Av Ana de Viya, 21, 11009 Cádiz, Spain; Hospital Sanitas La Moraleja, Av de Francisco Pi y Margall, 81, 28050 Madrid, Spain; Hospital Universitario Puerta de Hierro Majadahonda, Calle Manuel de Falla, 1, 28222 Majadahonda, Madrid Spain; Hospital Virgen del Puerto, Paraje Valcorchero, 10600 Plasencia, Cáceres Spain; Hospital Virgen de la Macarena, Avd. Dr. Fedriani, 3, 41071 Sevilla, Spain; Hospital Universitario de Salamanca, Paseo San Vicente, 182, 37007 Salamanca, Spain

**Keywords:** Prevalence, Fracture, Rheumatoid arthritis, Systemic lupus erythematosus, Glucocorticoid

## Abstract

**Background:**

Glucocorticoid (GC) therapy is associated with an increased risk of fractures. The main objective of this study was to determine the prevalence of undiagnosed vertebral fractures in women chronically using GC therapy for autoimmune disorders. We also determined the prevalence of non-vertebral fractures, and investigated whether factors such as quality-of-life and future fracture risk are associated with vertebral/non-vertebral fractures.

**Methods:**

This was a multicenter cross-sectional study conducted in Spain. All women had rheumatoid arthritis (RA) and/or systemic lupus erythematosus (SLE). Radiological morphometric vertebral fractures were evaluated centrally (Genant semiquantitative method), whereas non-vertebral fractures were not assessed by radiography. Before radiography, patients were asked whether they had vertebral/non-vertebral fractures, hereafter referred to as ‘self-reported’ fractures. Assessment tools included the Disease Activity Score (DAS28), the SF-36 questionnaire, and FRAX®.

**Results:**

Complete data were obtained for 576 outpatients with RA and/or SLE (83.3 % had RA); mean [SD] age 59.6 [15] years. Of all patients, 6.4 % had self-reported vertebral fractures, whereas 18.9 % had morphometric vertebral fractures (RA: 7.1 % self-reported vs. 20.0 % morphometric; SLE: 3.2 % self-reported vs. 13.7 % morphometric). Non-vertebral fractures were self-reported by 9.8 % of RA and 5.3 % of SLE patients. Low physical functioning was associated with morphometric vertebral fractures (mean [SD] SF-36 score 18.8 [6.0] when present vs. 20.1 [5.9] when absent; *p* = 0.028) and self-reported non-vertebral fractures (16.7 [5.2] when present vs. 20.1 [5.9] when absent; *p* < 0.001). Mean [SD] DAS28 was higher (*p* = 0.013) when any self-reported fractures were present (4.0 [1.3]) than absent (3.6 [1.3]). Based on FRAX® analysis, patients with vs. without morphometric vertebral fractures had higher 10-year probabilities of major osteoporotic fractures (mean [SD] 17.9 [12.9]% vs. 9.9 [9.6]%; *p* < 0.001) and hip fractures (11.0 [11.7]% vs. 4.6 [8.1]%; *p* < 0.001).

**Conclusions:**

Morphometric vertebral fractures were detected in 18.9 % of patients, i.e. 3-times more frequently than verbally reported by patients. Patients with vs. without fractures had worse quality-of-life and increased fracture risk. Accordingly, it is of utmost importance that women chronically using GCs are assessed for fractures, including morphometric vertebral fractures.

## Background

Glucocorticoid (GC) medications are widely used to treat various inflammatory and autoimmune disorders, including rheumatoid arthritis (RA) and systemic lupus erythematosus (SLE). Community surveys indicate that 0.2 to 0.5 % of the general population may receive GC therapy [1]. However, GC therapy is associated with increased risk of fractures, elevated by as much as 75 % within the first 3 months of treatment [2], and rapid decrease in bone mineral density (BMD) and trabecular bone volume (TBV), e.g. a 25 % reduction in TBV has been observed after 5–7 months of GC therapy [3]. While GC treatments are the most common cause of secondary osteoporosis [1], they may also increase the risk of fractures by triggering deterioration of bone quality. This may explain why Van Staa et al. [4] demonstrated that postmenopausal women taking GCs had more than twice the risk of fracture versus women not taking GCs, even though the GC users had higher BMD than the controls.

With regard to the type of fractures that commonly occur in patients using GC therapy and/or diagnosed with RA/SLE, vertebral fractures are of particular interest. This is not only due to the high prevalence of vertebral fractures in patients with RA (15–36 %) [5–8], and in patients with SLE (20-50 %) [9–13], but also the great impact that these fractures have on quality-of-life (QoL; including physical functioning) [5, 14, 15], mortality [16–18], and the risk of future fractures [19]. Patients with vertebral fractures may also suffer from long-term back pain [20]. It is therefore notable that vertebral fractures are often asymptomatic or mild-to-moderately symptomatic [2, 21, 22]; thus, under-diagnosis [9, 22, 23] and under-treatment [24] are major problems. For example, vertebral fractures were detected by X-rays in 14.1 % of 934 women admitted to hospital (for reasons not related to osteoporosis), whereas only 1.8 % of the 934 patients were diagnosed with vertebral fractures without X-ray analysis [25]. Similarly, Angeli et al. [21] found that ultrasound and BMD measurements were ineffective for predicting the number and severity of vertebral fractures in women undergoing GC therapy; thus, appropriate diagnostic procedures and physician awareness of the risk of fractures are paramount to identify and treat patients at high risk [2].

Few studies have investigated the prevalence of fractures in Spanish patients receiving chronic GC therapy. Accordingly, the primary objective of this study was to determine the prevalence of undiagnosed vertebral fractures in women chronically using GC therapy for autoimmune disorders in Spain. To meet this objective, patients were asked whether or not they had any vertebral fractures, hereafter referred to as ‘self-reported’ fractures, and their prevalence was compared to the prevalence of vertebral fractures subsequently identified by radiography. The key secondary objective was to determine the prevalence of self-reported non-vertebral fractures.

## Methods

### Patients

#### Inclusion criteria

Female outpatients aged ≥18 years, diagnosed with RA and/or SLE for more than 1 year, taking GC treatment (dose ≥2.5 mg of prednisone, or equivalent) for at least 3 months. At least 4 of 7 criteria had to be present for a diagnosis of RA [26], whereas at least four of 11 criteria had to be present for a diagnosis of SLE [27]. Patients had to be willing to undergo lateral thoracic and lumbar spine X-rays.

#### Exclusion criteria

The most clinically relevant exclusion criteria were: a diagnosis of metabolic bone disease, excluding osteoporosis, but including any active neoplastic disease; pregnancy at the time of radiological assessment. The other exclusion criteria were: the patient being an employee of the study sponsor, or being investigator site personnel directly affiliated with the study or their immediate family (spouse, parent, child, or sibling); current enrollment in or discontinuation from a clinical trial in the last 30 days, or concurrent enrollment in any other type of medical research that was judged as not being scientifically or medically compatible with this study.

### Objectives

The primary objective of this study was to determine the prevalence of undiagnosed vertebral fractures in women receiving chronic GC therapy. The key secondary objective was to determine the prevalence of self-reported non-vertebral fractures in these patients. Other secondary objectives were to assess potential relationships between the presence/absence of fractures and the following variables: patient characteristics; the presence of other diseases affecting bone metabolism; cumulative GC dose (prednisone 5 mg or equivalent for other GCs, received during the last 10 years, including intravenous pulses); disease activity and disability with RA, using the Disease Activity Score (DAS28) [28], and the Spanish 20-item Health Assessment Questionnaire (HAQ) [29]; Health-Related Quality-of-Life (HRQoL), using the SF-36 questionnaire; the 10-year risk of a major osteoporotic fracture or a hip fracture, using the fracture risk-assessment tool (FRAX®, available at http://www.shef.ac.uk/FRAX/); treatments to reduce bone loss.

### Study design

This was a population-based, cross-sectional, outpatient study conducted at 28 centers in Spain between June 2010 and July 2011. In order to have a widespread sample of the Spanish patient population, the participating sites were proportionally selected from Autonomous Communities throughout Spain, according to overall population figures published by the National Statistics Institute (INE) in January 2008. The study was approved by the responsible institutional review board at each study site,^1^ and was conducted in accordance with the ethical principles of the Declaration of Helsinki, good clinical practice guidelines, and applicable laws and regulations.

Patients attended their rheumatologist’s office for any reason, and following informed consent could be included in this study. Each rheumatologist recruited patients consecutively. All data that had to be verbally collected from the patient, i.e. on the nature of vertebral and non-vertebral fractures, and on HRQoL, as well as X-rays, were collected during this single visit at the rheumatologist’s office.

### Fracture assessments

Vertebral fractures were detected using X-rays, evaluated by a central reader using the Genant semiquantitative method [30]. A morphometric radiological vertebral fracture was defined as at least a 20 % reduction (at least 4 mm) in anterior, middle, and/or posterior vertebral height, and a change of at least a 1-degree angle according to the semiquantitative assessment. According to semiquantitative endpoints, the severity of each vertebral fracture is defined as grade 0 (no fracture), grade 1 (mild), grade 2 (moderate), or grade 3 (severe), based on the reduction in vertebral height. Normal vertebrae (grade 0) show minimal deformity, with a <20 % reduction in anterior, middle, and posterior vertebral height. Mild vertebral deformity (grade 1) is defined as a reduction of 20–25 % in vertebral height. Moderate (grade 2) and severe (grade 3) vertebral fractures are respectively defined as reductions of 25–40 % and >40 % in vertebral height.

Before radiography, the rheumatologists asked their patients whether or not they had any vertebral or non-vertebral fractures (the latter in the proximal femur, humerus, distal radius, sternum/ribs, tibia, pelvis, diaphyseal femur, distal femur); we refer to these as ‘self-reported’ fractures.

### Statistical analysis

To estimate the prevalence of vertebral and non-vertebral fractures with a precision of ±3.5 %, expressed as 95 % confidence intervals (CIs), and with a significance level of 5 %, a study population of 587 patients was needed. Assuming that 5 % of the sample would be non-evaluable due to screening failures, the minimum sample size for screened patients was 618 patients.

#### Primary analysis

The number and percentage of women with morphometric vertebral fractures were determined overall (i.e. for all patients) and by diagnosis (i.e. RA or SLE), based on X-ray data. The number and percentage of women with self-reported vertebral fractures were also summarized overall and by diagnosis. However, the total number of vertebral fractures, their location (lumbar/thoracic), and severity (mild/moderate/severe) were only determined using X-ray data, and not based on self-reports.

#### Key secondary analysis

The number and percentage of patients with, and the total number of, self-reported non-vertebral fractures were summarized overall, by diagnosis, and by fracture location (e.g. distal radius, proximal femur, rib).

Statistical methods, used to compare the following variables in the presence and absence of vertebral and/or non-vertebral fractures, included: *t*-test for DAS28, HAQ, FRAX®, and HRQoL; Kruskal-Wallis test for non-normally distributed variables (patient demographics and cumulative GC dose); and, as a supportive analysis for cumulative GC dose, analysis of covariance (ANCOVA) adjusted for 9 covariates. The ANCOVA covariates were selected by medical judgment, and comprised: age, body mass index (BMI), menopausal status, diagnosis (RA or SLE), presence of disease affecting bone metabolism, exposure to therapies associated with risk of fracture, current GC dose, smoking status, and whether the patient’s parent had sustained a hip fracture. All statistical tests are exploratory, and significance was set at *p* < 0.05 (2-sided). No imputations of missing values and no multiplicity adjustments were performed. Data were analyzed using SAS software^©^ version 8.2.

## Results

### Patient disposition and baseline demographics

**Fig. 1:**
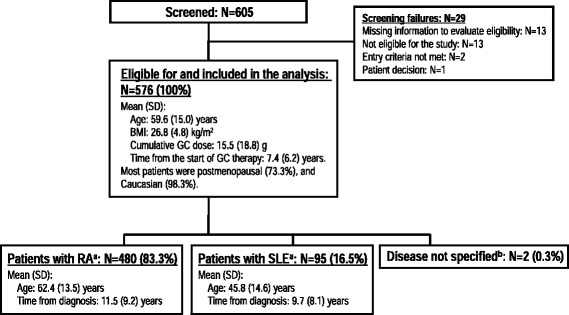
Patient disposition and baseline demographics. BMI: body mass index; GC: glucocorticoid; N: total number of patients in group; RA: rheumatoid arthritis; SD: standard deviation; SLE: systemic lupus erythematosus. ^a^ 1 patient was diagnosed with RA and SLE and is included in both groups. ^b^ 2 patients were diagnosed with RA and/or SLE, although this diagnostic information was missing when the data was analyzed. These 2 patients were included in the analysis

Of the 605 screened outpatients, 576 met all eligibility criteria and were included in this cross-sectional study. One patient was withdrawn due to physician decision before being X-rayed, although their data were included in the self-reported fracture analysis. As shown in Fig. 1, 480 (83.3 %) patients had RA and 95 (16.5 %) patients had SLE. The patients with RA were older (mean [SD] age 62.4 [13.5] years) than the patients with SLE (45.8 [14.6] years),^2^ while the time from diagnosis was similar in the 2 groups (Fig. 1).

### Prevalence of vertebral fractures

**Fig. 2:**
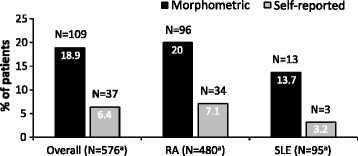
Estimated proportions of patients with vertebral fractures. N: total number of patients in group; RA: rheumatoid arthritis; SLE: systemic lupus erythematosus. ^a^ Total number of patients with/without fractures

As expected, the prevalence of morphometric vertebral fractures was higher than the prevalence of self-reported vertebral fractures in patients with RA, SLE, and overall (i.e. for all patients). Specifically, the numbers (proportion [%], 95 % CIs) of patients with at least 1 morphometric vertebral fracture vs. self-reported vertebral fracture were: 109 (18.9 %, 15.8-22.4) vs. 37 (6.4 %, 4.6–8.7) overall, 96 (20.0 %, 16.5–23.9) vs. 34 (7.1 %, 5.0–9.8) in RA patients, and 13 (13.7 %, 7.5–22.3) vs. 3 (3.2 %, 0.7–9.0) in SLE patients^2^ (Fig. 2). Only 3 of the 37 patients with self-reported vertebral fractures did not have these fractures confirmed in the morphometric radiological assessments. In the morphometric radiological assessments, 235 vertebral fractures were detected in the 109 patients; the locations and severity of these fractures were similar in patients with RA vs. those with SLE (Table 1).

**Table 1:** The prevalence of morphometric vertebral fractures and self-reported non-vertebral fractures

	Overall	Patients with RA	Patients with SLE
	*N* = 576	*N* = 480	*N* = 95
Vertebral fractures:			
Patients (%) with at least 1 vertebral fracture	109 (18.9)	96 (20.0)	13 (13.7)
Number of vertebral fractures (%)	235 (100.0)	203 (100.0)	32 (100.0)
Thoracic	159 (67.7)	135 (66.5)	24 (75.0)
Lumbar	76 (32.3)	68 (33.5)	8 (25.0)
Mild	124 (52.8)	105 (51.7)	19 (59.4)
Moderate	76 (32.3)	68 (33.5)	8 (25.0)
Severe	35 (14.9)	30 (14.8)	5 (15.6)
Non-vertebral fractures:			
Patients (%) with at least 1 non-vertebral fracture	52 (9.0)	47 (9.8)	5 (5.3)
Number of non-vertebral fractures (%)	68 (100.0)	59 (100.0)	9 (100.0)
Proximal femur	14 (20.6)	13 (22.0)	1 (11.1)
Proximal humerus	8 (11.8)	8 (13.6)	0
Distal radius	19 (27.9)	17 (28.8)	2 (22.2)
Rib	7 (10.3)	4 (6.8)	3 (33.3)
Tibia	4 (5.9)	3 (5.1)	1 (11.1)
Pelvis	5 (7.4)	5 (8.5)	0
Other^a^	11 (16.2)	9 (15.3)	2 (22.2)

*N* total number of patients in group, *RA* rheumatoid arthritis, *SLE* systemic lupus erythematosus

^a^No fractures were reported in the distal femur, diaphyseal femur, or sternum

### Patient characteristics by morphometric vertebral fracture status

Patients with morphometric vertebral fractures (*N* = 109) were statistically significantly older (mean [SD] 69.7 [10.8] vs. 57.3 [14.9] years) than those without fractures (*N* = 467), had a longer time since menopause (22.4 [10.1] vs. 16.7 [10.5] years), and shorter height (155.9 [7.6] vs. 158.7 [7.0] cm) (*p* < 0.001 for all 3 parameters; Table 2).

**Table 2:** Summary of demographics, glucocorticoid dose, and treatments to reduce bone loss^a^ for patients with or without morphometric vertebral fractures

Parameter	Vertebral fractures		
	Overall	Present	Absent
	*N* = 576	*N* = 109	*N* = 467
Age, years			
Mean (SD)	59.6 (15.00)	69.7 (10.81)	57.3 (14.88)
P-value^b^		<0.001	
BMI, kg/m^2^			
Mean (SD)	26.8 (4.83)	26.6 (3.61)	26.9 (5.08)
*P*-value^b^		0.724	
Race, n (%)			
Caucasian	566 (98.3)	109 (100)	457 (97.9)
Black	3 (0.5)	0 (0.0)	3 (0.6)
American Indian or Alaska native	5 (0.9)	0 (0.0)	5 (1.1)
Other	2 (0.3)	0 (0.0)	2 (0.4)
Menopause, n (%)			
Started	422 (73.3)	104 (95.4)	318 (68.1)
Started at age ≤40	37 (6.4)	11 (10.1)	26 (5.6)
Not started	150 (26.0)	4 (3.7)	146 (31.3)
Unknown	4 (0.7)	1 (0.9)	3 (0.6)
Time since menopause, years			
Mean (SD)	18.1 (10.66)	22.4 (10.06)	16.7 (10.47)
P-value^b^		<0.001	
Height, cm			
Mean (SD)	158.2 (7.20)	155.9 (7.61)	158.7 (7.00)
P-value^b^		<0.001	
Cumulative GC dose, g			
Mean (SD)	15.5 (18.77)	18.3 (23.00)	14.9 (17.63)
P-value^b^		0.346	
Time from the start of GC use, years			
Mean (SD)	7.4 (6.15)	8.4 (7.60)	7.1 (5.74)
P-value^b^		0.279	
Percentage of patients taking calcium and/or vitamin D	49.3	52.3	48.6
Calcium	45.0	49.5	43.9
Vitamin D	45.0	51.4	43.5
Percentage of patients taking bisphosphonates	42.7	66.1	37.3
Alendronate	20.3	32.1	17.6
Risedronate	16.5	22.9	15.0
Ibandronate	9.7	19.3	7.5
Zoledronic acid	1.2	2.8	0.9
Percentage of patients taking other medications			
Strontium ranelate	2.3	3.7	1.9
Teriparatide	1.9	6.4	0.9
Raloxifine	1.2	2.8	0.9

*BMI* body mass index, *GC* glucocorticoid, *n* number of patients, *N* total number of patients in group, *SD* standard deviation

^a^Treatments to reduce bone loss used by ≥1.2 % of the overall population

^b^ *P*-value is for the difference between the 2 fracture cohorts, calculated using Kruskal-Wallis test

### Prevalence of self-reported non-vertebral fractures

The prevalence of patients with at least 1 self-reported non-vertebral fracture^2^, and the number and locations of self-reported non-vertebral fractures in these patients, are shown in Table 1. For RA, the most common locations of self-reported non-vertebral fractures were: 17 (28.8 %) in the distal radius, and 13 (22.0 %) in the proximal femur. For SLE, the most common self-reported non-vertebral fracture locations were 3 (33.3 %) in the rib, and 2 (22.2 %) in the distal radius.

### Diseases affecting bone metabolism

Similar proportions of patients with versus without morphometric vertebral fractures, or with versus without self-reported non-vertebral fractures, had at least 1 disease affecting bone metabolism. Specifically, 7 (6.4 %) of 109 patients with and 29 (6.2 %) of 467 patients without morphometric vertebral fractures, and 3 (6.5 %) of 46 patients with and 33 (6.3 %) of 530 patients without self-reported main non-vertebral fractures had at least 1 disease affecting bone metabolism. Diseases affecting bone metabolism in >1 % of patients were: secondary amenorrhea (duration >1 year), hyperthyroidism, type I diabetes, liver disease, and urolithiasis.

### Cumulative GC dose

In patients with versus without morphometric vertebral fractures, cumulative GC dose and time from the start of GC use were not significantly different, despite being numerically higher (Table 2). Similarly, following adjustment for selected baseline variables,^3^ no statistically significant difference (*p* = 0.087) was detected in cumulative GC dose in the presence versus absence of morphometric vertebral fractures; however, cumulative GC dose was about 40 % higher (*p* = 0.026) for patients with versus those without self-reported vertebral fractures, after the adjustment.

In the presence versus absence of any self-reported main non-vertebral fractures, cumulative GC dose was higher (mean [SD] 28.6 [32.1] vs. 14.4 [16.7] g; *N* = 46 vs. *N* = 527; *p* < 0.001), and time from the start of GC use was longer (12.8 [8.7] vs. 6.9 [5.6] years; *N* = 46 vs. *N* = 528; *p* < 0.001). Likewise, following adjustment for selected baseline variables^3^, cumulative GC dose was about 70 % higher (*p* < 0.001) in patients with versus those without any self-reported main non-vertebral fractures.

### Relationships between RA and fracture status

**Fig. 3:**
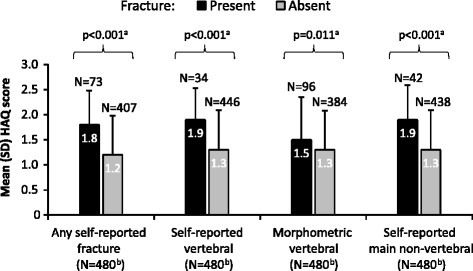
Disability vs. fracture status in patients with RA. HAQ: Spanish 20-item Health Assessment Questionnaire; N: total number of patients in group; RA: rheumatoid arthritis; SD: standard deviation. ^a^ Statistically significantly different mean HAQ scores by fracture status, calculated using *t*-test. ^b^ Total number of RA patients with/without fractures

DAS28 scores were higher (mean [SD] 4.0 [1.3] vs. 3.6 [1.3]; *p* = 0.013) when any self-reported fractures were present (*N* = 73) than in their absence (*N* = 407), indicating that RA was more active in the presence of self-reported fractures. Similarly, the presence of self-reported fractures (i.e. any fractures, vertebral fractures, main non-vertebral fractures; *p* < 0.001 for all 3 groups) was associated with greater disability, as shown by higher HAQ scores than in RA patients without fractures (Fig. 3). The presence of morphometric vertebral fractures was also associated with greater disability in RA patients than in the absence of fractures (*p* = 0.011), although less so than for patients with self-reported vertebral fractures (Fig. 3).

### Health-related quality-of-life

SF-36 physical component scores were statistically significantly lower for patients with versus without self-reported vertebral fractures (mean [SD] 30.5 [16.5] vs. 41.3 [19.8]; *N* = 37 vs. *N* = 536; *p* < 0.001), and also for patients with versus without self-reported main non-vertebral fractures (mean [SD] 31.7 [21.4] vs. 41.4 [19.4]; *N* = 46 vs. *N* = 527; *p* < 0.001). Of the SF-36 subscores for the physical component, low physical functioning and role limitation due to physical problems were both statistically significantly associated with self-reported vertebral fractures and self-reported main non-vertebral fractures (data not shown). Of all SF-36 scores, only the low physical functioning SF-36 subscore was statistically significantly associated with morphometric vertebral fractures, with lower scores in their presence versus absence (mean [SD] 18.8 [6.0] vs. 21.1 [5.9]; *N* = 108 vs. *N* = 465; *p* = 0.028).

### Ten-year risk of future fractures

In the FRAX® analysis, patients who already had a morphometric or self-reported vertebral fracture, or self-reported non-vertebral fracture, had statistically significantly higher 10-year probabilities of sustaining a major osteoporotic fracture or hip fracture than patients without fractures. For example, mean (SD) risk of sustaining a major osteoporotic fracture was 17.9 % (12.92) for patients with morphometric vertebral fractures (*N* = 106) vs. 9.9 % (9.64) for patients without these fractures (*N* = 469; *p* < 0.001).

### Treatment to reduce bone loss

Higher proportions of patients with versus without morphometric vertebral fractures (81.7 % vs. 63.2 %), self-reported vertebral fractures (97.3 % vs. 64.6 %), and self-reported main non-vertebral fractures (95.7 % vs. 64.2 %) were receiving treatment to reduce bone loss. For each type of fracture, the main treatments used to reduce bone loss were vitamin D, calcium, bisphosphonates, strontium ranelate, teriparatide, and raloxifine. The proportions of patients with versus without morphometric vertebral fractures using these medications are shown in Table 2. The proportions of patients with versus without self-reported vertebral fractures receiving medication were 89.2 % vs. 39.5 % (bisphosphonates), 52.3 % vs. 48.6 % (vitamin D and/or calcium), 16.2 % vs. 0.9 % (teriparatide), 8.1 % vs. 0.7 % (raloxifen), and 5.4 % vs. 2.0 % (strontium ranelate). The proportions of patients with versus without self-reported main non-vertebral fractures receiving medication were 82.6 % vs. 39.2 % (bisphosphonates), 65.2 % vs. 47.9 % (vitamin D and/or calcium), 10.9 % vs. 1.1 % (teriparatide), 6.5 % vs. 0.8 % (raloxifen), and 10.9 % vs. 1.5 % (strontium ranelate).

## Discussion

In this population-based, cross-sectional study, centralized X-ray assessment increased the detection rate of vertebral fractures to 18.9 %, from 6.4 % for self-reported vertebral fractures, in women with RA and/or SLE receiving chronic GC therapy. This finding is consistent with other reports suggesting that vertebral fractures are under-diagnosed in post-menopausal women [21, 23, 25]. These reports included a cross-sectional study by Angeli et al. [21] in which patients (*N* = 551) received chronic GC therapy, and had RA, SLE, asthma/chronic obstructive pulmonary disease, rheumatic polymyalgia, or another vasculitis or connective tissue disease. Moreover, in a sample of 2451 post-menopausal women with osteoporosis, in the IMPACT study, under-diagnosis of vertebral fractures was shown to be a worldwide problem (false-negative rates: North America, 45.2 %; Latin America, 46.5 %; Europe/South Africa/Australia, 29.5 %) [23].

In our study, 85.1 % of vertebral fractures detected in centrally assessed X-rays were mild-to-moderate in severity, compatible with the high prevalence (37 %) of asymptomatic vertebral fractures in GC-users reported by Angeli et al. [21]. In addition, in our study, vertebral and non-vertebral fractures had a detrimental impact on patients’ lives, relative to patients without these fractures. This included greater disability and reduced physical functioning in women with RA and/or SLE who had self-reported or morphometric fractures. Likewise, RA/SLE patients with self-reported or morphometric vertebral fractures had a higher 10-year risk of suffering from a major osteoporotic fracture or hip fracture, compared with patients without fractures, as determined by FRAX® analysis. A similar finding of increased 10-year risk of fractures in patients with SLE versus healthy controls was recently reported by Mak et al. [31].

In this study, the impact of fractures on disability and role limitation due to physical problems, and the 10-year risk of fracture, appeared to be particularly high for patients with self-reported vertebral fractures versus morphometric vertebral fractures. Together with the higher prevalence of morphometric than self-reported vertebral fractures (18.9 % vs. 6.4 %), overall these data suggest that the morphometric fractures may have been milder than the self-reported fractures.

Vertebral fractures appeared to be prevalent in RA and SLE patients, being detected in 20 % of patients with RA and 13.7 % of patients with SLE by centralized X-ray assessment. Although, as the RA patients were older than the SLE patients, and age influences fracture risk, no direct comparison of fracture prevalence should be made between these 2 patient groups, and these data should only act as an indicator of the high prevalence of vertebral fractures in these patients. Similarly high prevalence rates of vertebral fractures, detected in X-rays, have previously been found in studies of RA (15–36 % of 97–275 patients) [5–8] and SLE (20–50 % of 52–210 patients) [9–13]. The number of patients with self-reported non-vertebral fractures in our cross-sectional study (found in 9.8 % of RA and 5.3 % of SLE patients) also appeared to be consistent with previous studies of RA [8, 32], including a reported prevalence of 16 % in 102 RA patients (determined via interviews, and by checking past radiological reports and chart review) [8]. However, Ørstavik et al. [33] did suggest that, with the likely exception of hip fractures, “non-vertebral fractures do not seem to be a substantial burden in RA”.

With regard to factors that could cause/increase the risks of fractures, RA on its own may be a risk factor [5, 7, 34], although GC administration is also said to be the leading cause of secondary osteoporosis [1]. Thus, it is notable that Angeli et al. [21] concluded that RA probably contributes to the risk of fractures, although these researchers could not dissect the potential impact of RA versus GC therapy on risk of fracture, while Van Staa et al. [32] stated that the increased risk of fracture is due to a combination of RA activity and GC therapy. Indeed, inherent covariates make it difficult to attribute an increased risk of fractures to GC usage in our study, e.g. patients with morphometric vertebral fractures had a statistically significantly longer time since menopause than patients with an absence of these fractures. Similarly, RA activity was higher in the presence of any self-reported fractures than in their absence, although other diseases affecting bone metabolism (e.g. secondary amenorrhea, hyperthyroidism) were present in similar proportions of patients (6.2–6.5 %) with and without morphometric vertebral and self-reported non-vertebral fractures. Thus, other diseases affecting bone metabolism were unlikely to complicate these analyses.

Both before and after adjusting for various covariates, including age and RA and SLE diagnoses, patients with any self-reported main non-vertebral fractures had a higher cumulative GC dose than those without these fractures. Thus, chronic GC therapy does appear to be associated with an increased risk of any self-reported main non-vertebral fractures in our population of patients with RA and/or SLE.^4^ These results are particularly significant in terms of FRAX® as, at present, this tool does not consider any potential impact of cumulative or current GC dose on the risk of fracture, and only collects data about whether or not the patient uses steroids. Therefore, predictions of fracture risk using FRAX® may be improved by considering the chronicity and dosage of GC therapy. In addition, the links between chronicity and dosage of GC therapy and increased risk of non-vertebral fractures in our study are similar to results in other publications [35, 36] including an extensive meta-analysis [37]. Nevertheless, our results are inconclusive with regard to any impact of cumulative GC dose on the risk of sustaining a vertebral fracture. Conclusions also differ between published studies as to whether or not cumulative GC dose is associated with the risk of sustaining a vertebral fracture [4, 21, 37, 38].

With regard to potential mechanism(s) linking GC therapy with increased risk of nonvertebral fractures in our study, it is possible that GC therapy does not only increase fracture risk by reducing BMD [4, 21, 31, 39, 40], but also by reducing bone quality through microarchitectural changes [40]. This may explain why, in our study, a higher proportion of patients suffered morphometric vertebral fractures, and/or self-reported main non-vertebral fractures, when taking treatments to reduce bone loss than when not taking these treatments. Thus, ideally, prophylactic treatments should be administered to improve bone quality as well as quantity. Similarly, it may be prudent to radiologically assess patients at high risk of vertebral fractures, as recently done by Clark et al. [41], where high risk patients were identified using a novel primary care-based screening tool. The resulting radiological analyses could be performed in conjunction with the Genant semiquantitative method used in our study and other studies of RA [8, 32] and SLE [10, 14, 22].

Our study does have some limitations. Firstly, no control group was included. Thus, direct comparisons of, for instance, the prevalence of fractures could not be made between patients using GCs versus those not using GCs, and any indirect comparisons with other studies are complicated by potential differences in study design and patient populations. Secondly, all of the non-vertebral fractures were only self-reported. Thirdly, higher proportions of patients who had fractures were receiving medications to reduce bone loss, versus patients without fractures. Thus, while these medications may have reduced the prevalence of fractures, and may be regarded as a limitation when determining the prevalence of fractures in patients with RA/SLE, this was in a real-world clinical population. Moreover, our study does have several strengths, including a large sample size relative to comparable studies [7, 8, 12, 13, 33], and centralized reading of the X-rays with uniform criteria that was likely to limit variability in the dataset. This thorough approach is likely to have increased the accuracy of the estimated prevalence of vertebral fractures, thus allowing more accurate determination of the impact of these fractures on patients’ physical functioning and overall QoL.

## Conclusions

In summary, we detected a higher rate of vertebral fractures in women with RA and/or SLE, chronically treated with GCs, in centrally assessed X-rays when compared with self-reported fractures. Moreover, vertebral and non-vertebral fractures appeared to be very prevalent in these patients. Our results also suggest that non-vertebral fractures may be related to chronic GC use, rather than solely due to underlying disease. However, our results are inconclusive with regard to any impact of cumulative GC dose on the risk of sustaining a vertebral fracture. In addition, vertebral and non-vertebral fractures had negative impacts upon QoL, particularly physical functioning, and increased the risk of further fractures in a 10-year period. It is therefore important to carefully monitor patients with RA and/or SLE who receive chronic GC therapy, to detect fractures including morphometric vertebral fractures. Also, in our opinion, physicians should implement preventative measures in patients with RA or SLE who receive chronic GC therapy, to decrease the risk of sustaining vertebral and non-vertebral fractures.

## Footnotes

1 The institutional review boards for the 28 study sites were: Hospital de Lugo, Lugo; Hospital Puerta del Mar, Cádiz; Hospital Sanitas La Moraleja, Madrid; Hospital Universitario Puerta de Hierro Majadahonda, Madrid; Hospital Virgen del Puerto, Plasencia; Hospital Virgen de la Macarena, Sevilla; Hospital Universitario de Salamanca, Salamanca.

2 It was not an a priori objective of this study to compare the prevalence of fractures in patients with RA versus patients with SLE.

3 The selected baseline variables were: age, BMI, menopausal status, diagnosis (RA and/or SLE), presence of disease affecting bone metabolism, exposure to therapies associated with risk of fracture, current GC dose, smoking status, and whether the patient’s parent had sustained a hip fracture.

4 These analyses were not performed separately for RA or SLE subgroups, as these analyses were not planned, and SLE subgroup analysis could not be done retrospectively due to the small number of SLE patients.
